# The Role of Simulation Science in Public Health at the Agency for Toxic Substances and Disease Registry: An Overview and Analysis of the Last Decade

**DOI:** 10.3390/toxics12110811

**Published:** 2024-11-12

**Authors:** Siddhi Desai, Jewell Wilson, Chao Ji, Jason Sautner, Andrew J. Prussia, Eugene Demchuk, M. Moiz Mumtaz, Patricia Ruiz

**Affiliations:** 1 Oak Ridge Institute for Science and Education, Oak Ridge, TN 37830, USA; 2 Office of Innovation and Analytics, Agency for Toxic Substances and Disease Registry, Atlanta, GA 30329, USA; 3 Office of Associate Director for Science, Agency for Toxic Substances and Disease Registry, Atlanta, GA 30329, USA

**Keywords:** computational toxicology, PBPK, QSAR, BMD, fate and transport modeling, machine learning modeling, simulation sciences, predictive toxicology

## Abstract

Environmental exposures are ubiquitous and play a significant, and sometimes understated, role in public health as they can lead to the development of various chronic and infectious diseases. In an ideal world, there would be sufficient experimental data to determine the health effects of exposure to priority environmental contaminants. However, this is not the case, as emerging chemicals are continuously added to this list, furthering the data gaps. Recently, simulation science has evolved and can provide appropriate solutions using a multitude of computational methods and tools. In its quest to protect communities across the country from environmental health threats, ATSDR employs a variety of simulation science tools such as Physiologically Based Pharmacokinetic (PBPK) modeling, Quantitative Structure–Activity Relationship (QSAR) modeling, and benchmark dose (BMD) modeling, among others. ATSDR’s use of such tools has enabled the agency to evaluate exposures in a timely, efficient, and effective manner. ATSDR’s work in simulation science has also had a notable impact beyond the agency, as evidenced by external researchers’ widespread appraisal and adaptation of the agency’s methodology. ATSDR continues to advance simulation science tools and their applications by collaborating with researchers within and outside the agency, including other federal/state agencies, NGOs, the private sector, and academia.

## 1. Introduction

Environmental pollution, such as oil spill accidents and contaminated food, air, and water, raises significant concerns for public health. Ideally, experimental chemical testing would provide evidence of the potential effects of these environmental chemicals on human health. However, in most scenarios, the data we need are unavailable. Simulation science tools can be used to address environmental chemical exposure and its potential health effects, as well as related challenges [1,2,3,4,5,6,7]. Simulation science uses informatics, mathematical and statistical modeling, and computational toxicology to assess the potential health risks of chemical exposure. These tools can be used to break down complex environmental problems and inform decision making.

The Agency for Toxic Substances and Disease Registry (ATSDR), a federal public health agency of the U.S. Department of Health and Human Services, has played a crucial role in assessing and addressing the potential health risks associated with toxic substances. Like the Centers for Disease Control and Prevention (CDC), ATSDR’s primary objective is to protect public health by preventing and/or minimizing exposure to hazardous substances. At ATSDR, simulation science approaches have become essential tools in understanding the adverse effects of environmental exposures on human health. ATSDR utilizes sophisticated computational models, tools, and data analysis techniques to predict chemical toxicity, identify potential hazards, and prioritize risk assessment efforts.

ATSDR’s Office of Innovation and Analytics (OIA) is integral in the collection, analysis, and interpretation of data on exposure to hazardous substances. OIA further leverages its Simulation Science Section (SSS) in the provision of analytical and modeling expertise as well as the development of novel computational tools to support the Agency’s public health endeavors [8]. Subsequently, ATSDR and its partners are able to make actionable decisions regarding exposure to hazardous substances. Informed decision making has had a positive effect of public health [9].

**Figure 1:**
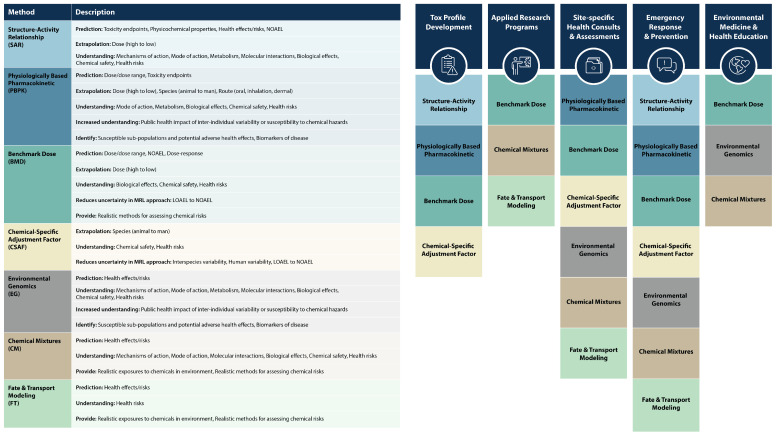
Simulation science methods: features and impacted areas.

Since establishing a state-of-the-art computational toxicology laboratory in 1998 (later renamed the ‘Simulation Science Section (SSS)’ within OIA), computational tools have been used in various aspects of human health risk assessment, as seen in Figure 1. These computational modeling approaches include the following:

- Physiologically Based Pharmacokinetic (PBPK) modeling;
- Quantitative Structure–Activity Relationship (QSAR) analysis;
- Computational systems biology;
- Benchmark dose (BMD) modeling;
- Fate and transport modeling.

PBPK models translate Points of Departure (PODs) from animal studies to the target human population by simulating chemical absorption, distribution, metabolism, and excretion. POD is the dose necessary to produce a particular effect of interest. QSAR predicts chemicals’ physiochemical, biological, and toxicological characteristics based on their molecular structures. QSAR bridges knowledge gaps when toxicological data are unavailable by screening chemicals to prioritize those that need more attention. Computational systems biology establishes associations between chemical exposures, gene expression, biological pathways, and potential health effects. BMD models help to estimate PODs by modeling the dose–response shapes of toxicology data. Fate and transport modeling reconstructs past chemical exposures through mathematical modeling.

ATSDR plays a crucial role in the risk assessment process by leveraging computational models, high-throughput screening data (HTS), and in vivo data. This enables the efficient identification of potentially hazardous chemical compounds that require more detailed investigation. ATSDR also provides timely recommendations for mitigating these public health risks. The information obtained through the use of simulation tools allows identifying vulnerable populations and devising targeted strategies to reduce exposure risks.

This paper presents an overview of the past decade of using computational tools for the risk assessment of toxic substances at ATSDR. These computational approaches include PBPK modeling, QSAR modeling, computational systems biology, BMD modeling, and fate and transport modeling. We discuss the modeling approaches, how they help meet data requirements, and how they assist in risk assessments for specific chemicals. Additionally, we summarize the collaborations used to address environmental health challenges using computational tools. Finally, we identify current challenges and gaps and offer insights into future directions for these computational tools.

## 2. Overview of Simulation Science Tools for Public Health

### 2.1. Physiologically Based Pharmacokinetic (PBPK) Modeling

One of ATSDR’s foremost goals is to determine tolerable exposure levels, or health guidance values (HGVs), for the numerous chemicals that people are regularly exposed to. HGVs are typically derived using all available data, including toxicological data from animal studies and epidemiological and biomonitoring data in humans. There are several limitations with this approach. One issue is that there is no upper limit on the number of studies required to produce the best HGV estimate. In practice, all pertinent available studies are used to support a calculated HGV. Additionally, translating exposure effects from animal studies to the target human populations conventionally requires extensive generalization, as indicated by the uncertainty factor (UF) values used in calculating minimal risk levels (MRLs) [10,11,12,13]. The UF values are designed to produce safe MRL values. However, they often overestimate tolerable exposure levels in humans, especially for sensitive populations such as pregnant women or the elderly.

PBPK modeling can be used to extrapolate toxicological information across different species, routes, and doses [14,15]. PBPK models estimate a chemical or mixture’s absorption, distribution, metabolism, and excretion. PBPK models simulate the human or animal body as a series of compartments (e.g., the lungs, fat, the brain, the liver, etc.) and track the plasma/blood flow through them [16,17]. PBPK modeling allows both cross-route and cross-duration extrapolation. For instance, chronic inhalation data can be used to derive an oral intermediate HGV via PBPK modeling. PBPK modeling can also eliminate the need for repeat animal and human studies that require extensive time and resource allocations for HGV estimation. Consequently, PBPK modeling greatly facilitates chemical risk assessment and benefits public health.

Given the advantages of PBPK modeling, ATSDR began developing a PBPK toolkit for high-priority chemicals. While there is an extensive number of PBPK models, they all vary in complexity and do not all use the same programming language. This makes routinely using them a challenge. ATSDR has been recoding highly advanced and/or efficient PBPK models, translating them onto a single platform (Berkeley Madonna), and modifying them to make them more generic. The resulting recoded models provide increased accessibility to public health assessors and can be more readily applied to the general population. Thus far, the ATSDR PBPK toolkit encompasses many chemicals, from heavy metals to dioxins and volatile organic compounds (VOCs).

The fidelity of each PBPK in the toolkit model has also been verified. Over a decade ago, ATSDR recoded models for cadmium, mercury, arsenic, and high-priority chemicals on the National Priority List [18,19]. The recoded cadmium model was validated by simulating urinary excretion data using dietary intake data from the 2003–2004 NHANES. The estimates closely matched actual urinary cadmium data [20]. Since then, the cadmium model has been used in various ways, ranging from use as a simple prototype to illustrate advancements in toxicokinetic modeling methods [21] to relatively in-depth summaries of its composition, function, and capabilities [22], and for comparison to more recently developed models [23]. The recoded cadmium model has been applied and adapted several times, demonstrating its effectiveness and far-reaching impact. The recoded cadmium model was used to estimate relative cadmium exposures in Thailand from diet and smoking in low- and high-exposure scenarios to evaluate the effectiveness of such models when applied to a more comprehensive exposure range [24]. The study used biomonitoring data (urinary cadmium) to estimate cadmium exposure, a method known as reverse dosimetry. The exposure estimates were noteworthy. Exposure estimates in the high-exposure area had a relatively small safety margin when compared to reference exposure levels. This emphasized the need to address cadmium exposures in the region because the metal is known to cause a myriad of health effects (ATSDR, 2012). More recently, researchers constructed one- and multi-compartment toxicokinetic models for cadmium geared toward the Chinese population. They used the recoded cadmium model [20] to characterize dietary cadmium intake as an age-dependent variable for the multi-compartment model. This enabled more accurate estimates of their intended population [25].

The PBPK toolkit has also incorporated recoded models for VOCs. In 2011, a generic, seven-compartment VOC model was recoded on Berkeley Madonna for use with six VOCs: benzene, carbon tetrachloride, dichloromethane, perchloroethylene, trichloroethylene, and vinyl chloride, and in 2012, another paper was published to present the progress towards developing and validating the generic VOC model [26]. The multi-compartment model was assessed by comparing its predictions with previously published human kinetic data and those of the original models from which it was derived. The generic model performed well in both cases (90% agreement). The VOC models have been primarily referenced in reviews and discussions of the current and potential advancements and limitations of PBPK models [27,28]. One article detailed federal agencies’ efforts to make PBPK models more accessible yet noted the lack of their widespread use in public health assessments [29]. Researchers in the Netherlands evaluated how the generic model [26] estimated blood dichloromethane concentrations in health participants [30]. The model helped determine first impressions during emergency risk assessments, although detailed applications would require a more sophisticated approach. The model was also used, with other strategies, to derive differential equations for a study creating a fuzzy number model of chloroform to inform swimming pool design and management [31]. The generic VOC model has propelled the creation and use of PBPK models and other computational tools in health risk assessments at the population level.

Dioxins and dioxin-like chemicals (DLCs) are another set of high-priority chemicals for ATSDR. In 2014, ATSDR recoded a model for 2,3,7,8-Tetracholorodibenzo-*p*-dioxin (TCDD), which is the most toxic congener of DLCs. ATSDR assessed its quality and concluded that the model may be helpful in health risk assessments given that it provides both age- and gender-related information about exposure as a function of its intake [32]. The recoded models have been adapted and used in various projects external to ATSDR, for example, in research to evaluate the toxicological risk of polychlorinated dibenzo-p-dioxins and dibenzofurans in the soil in Switzerland [33] and in a European Food Safety Authority (EFSA) scientific opinion article to assess the risk to humans and animals from dioxins in feed and food [34]. More recently, a study adapted the 2017 recoded model, among other things, to develop a dioxin mixture model [35]. This is a significant advancement, given that pollutants are usually encountered in mixtures. Thus, their interactions may play an important role in the overall toxicokinetic effects.

While the ATSDR’s human PBPK toolkit continues to grow, it has significantly influenced environmental pollutant risk assessment by assisting in extrapolating toxicological information across species, estimating a chemical or mixture’s absorption, distribution, metabolism, and excretion, and by allowing both cross-route and cross-duration extrapolation. While the recoded models have been intentionally made simple, they are constantly being adapted to produce more specific and wide-ranging models, fueling advancements in computational toxicology.

### 2.2. Quantitative Structure–Activity Relationship (QSAR) Modeling

QSAR modeling is extensively employed in public health and human risk assessment to predict the biological or toxicity activity or adverse effects of chemicals. These models assist in prioritizing chemicals for further testing, providing insights into their potential risks to human health [36,37,38,39].

QSAR models play a crucial role in reducing the need for animal testing by providing reliable predictions based on chemical structure information. They are particularly valuable when experimental data on chemicals are limited or lacking. While more experimental data could be collected, the extensive time and resources necessary for these studies may not be feasible.

We use this type of modeling in two ways: structure–activity relationship (SAR) models for structural alerts and QSAR models to quantify a chemical’s activity. SAR models are essentially qualitative as they predict whether the structural features of a compound indicate the potential to exhibit a specific health effect. QSAR models quantify these associations by predicting the chemical’s toxicological/biological activity. While they are used in various fields from agriculture to pharmaceutical industries, QSAR, as opposed to SAR, is of particular interest to health agencies. QSAR allows such agencies to bridge information gaps in toxicological data, thus eliminating the need for additional animal studies. In this way, it saves time and resources and enables users to establish HGVs efficiently.

However, despite the apparent benefits of using QSAR modeling, it has not yet been used to its potential. Not all models are created equal: the QSAR models possess varying predictive power and accuracy levels. Thus, developing, validating, and refining QSAR models is imperative as new experimental information becomes available to improve their accuracy. To this end, ATSDR is continually developing and validating QSAR models for major endpoints including endocrine disruption, carcinogenicity, mutagenicity, and developmental toxicity.

#### 2.2.1. Endocrine Disruption

ATSDR has expended significant efforts to develop and evaluate QSAR models for endocrine-disrupting chemicals. ATSDR researchers developed QSAR models to assess and define the relationship between molecular descriptors and the estrogenic potential of hydroxylated polychlorinated biphenyl (OH-PCB) metabolites. A follow-up study was published to illustrate estrogen receptor binding. The study expanded on the 2D QSAR approach of the earlier study using molecular docking and 3D QSAR techniques [40]. The 2D QSAR study has been referenced in various reviews and studies of computational modeling and endocrine disruptors [41,42,43,44,45]. More importantly, the study has been used to buttress the results and hypotheses of several investigations on the toxicology of PCBs. Baker and Bauer [46] (2015) investigated the chemotherapeutic effects of green tea by observing its impact on estrogen-sensitive breast cells that are induced to proliferate by an estrogenic PCB. They found that the PCB-102 effect was mediated by ERα (estrogen receptor alpha) and used the 2D QSAR study to confirm and reinforce their findings [46]. Additionally, researchers examining the presence of OH-PCBs in Arctic foxes in Svalbard used the 2D QSAR study to elucidate the toxicological implications of OH-PCB accumulation in the body [47]. Furthermore, Wang et al. [48] used the 2D QSAR study to state and explain features contributing to higher estrogenic activity. They explored OH-PCBs as Erβ (estrogen receptor beta) using 3D QSAR, molecular docking, and molecular dynamics.

The 3D QSAR study [40] has also significantly contributed to our mechanistic understanding of estrogenicity and breast cancer. It has underpinned hypotheses, results, and methods of multiple projects. For example, Ashtekar et al. [49] aimed to identify natural compounds that may inhibit HER2. HER2 plays a role in the development of breast cancer [49]. The 3D QSAR study was one of several papers used to clarify the pharmacophore modeling aspect of Ashtekar et al.’s study. In another publication, the 3D QSAR paper described the 2D and 3D models that Ashtekar’s research group developed [50]. Furthermore, the 3D QSAR study helped Farasani and Darbre emphasize the potential of triclosan (a PCB prevalent in personal care and household products) metabolites to have unknown toxic effects at the cellular level [51]. Notably, the study’s reach has not been limited to cancer research. The paper’s results were also used to emphasize the importance of investigating OH-PCBs in the marine environment [52,53] and in animal-derived food [54].

To further improve their models, ATSDR evaluated the QSAR approaches to investigate endocrine-disrupting chemicals. They compared the predictions of various QSAR models to experimental data for a large set of endocrine disruptors. This suggested consensus approach incorporating multiple models provided the most accurate predictions. The evaluation has been further analyzed in discussions of in silico methods and used to demonstrate the majority vote classifier method [55,56]. Additionally, Stanojević et al. used the study to justify their use of the consensus approach while computationally investigating the endocrine activity of biocidal active substances [57]. The consensus approach used by ATSDR researchers has encouraged other researchers to do the same, addressing the limitations of using single QSAR models to some extent.

ATSDR was also a part of a project to develop QSAR models for other endocrine-disrupting chemicals and androgen-active chemicals. As part of the project, researchers developed and validated five predictive classification models based on different algorithms. They amalgamated the models using two consensus approaches, majority vote, and convergent predictions. The researchers’ work has been credited several times, including in a paper on the latest high-throughput machine learning models for endocrine-disrupting chemicals [58]. The consensus approach developed for the project was also included in a review published by the Organization for Economic Cooperation and Development (OECD). The review highlighted the large training set used to create the models [59]. The consensus approach was further acknowledged as an effective way to circumvent the limitations of using singular QSAR models in a recent, extensive review of in silico studies on the nuclear receptor family [60].

#### 2.2.2. Carcinogenicity, Mutagenicity, and Developmental Toxicity

When evaluating the risk of exposure to chemicals via the environment, carcinogenicity, mutagenicity, and developmental toxicity are endpoints of significant interest. ATSDR researchers used a QSAR approach to understand the mutagenic and carcinogenic potential of specific PCB congeners and their metabolites. ATSDR’s work has supported hypotheses and conclusions drawn by other researchers. For instance, Koh et al. compared the serum levels of non-Aroclor PCBs in adolescents and their mothers living in East Chicago, Indiana, and Columbus Junction, Iowa [61]. They found certain PCBs were prevalent and, despite the resounding lack of toxicity data on one of the chemicals (PCB 35), the researchers could attribute potential mutagenicity and carcinogenicity to this specific congener. They did so using the approaches developed by ATSDR researchers. Furthermore, the findings reported by ATSDR researchers (such as higher mutagenic potential in lower chlorinated PCBs and their metabolites) have been broadly used to support several research projects. In some research studies, neurotoxic equivalent factor derivations were used to develop QSAR models for untested PCBs [62], and to expound on the properties of PCBs in Dickerson et al.’s preamble to investigating PCB-induced cell death mechanisms [63].

Furthermore, the PCB congeners’ work by ATSDR researchers indicated that mono- and di-chlorinated PCBs and their metabolites have a higher risk of genotoxicity effects and were further pursued through in vitro validation by Zhang et al. The authors studied a Chinese hamster-derived cell line expressing a human cytochrome enzyme that the researchers believed would transform PCBs to genotoxic metabolites. The enzyme appeared to convert certain PCBs to strong mutagens [64]. However, further work conducted by Zhang’s group suggested that certain tri- and tetra-chlorobiphenyls were more potent than the mono- and di-chlorinated congeners. [65]. Liu et al. account for this difference by pointing out that the S9-mediated assays used to generate the part of the database used in the ATSDR study would be biased against bigger molecules, such as more chlorinated biphenyls, inadvertently discounting their toxic potential.

ATSDR researchers also used QSAR modeling tools to investigate four ethylene glycol ethers (EGEs) and their metabolites for potential mutagenicity, carcinogenicity, and developmental toxicity. Reverse QSAR (rQSAR) was also used to identify structures that may induce developmental toxicity [66]. Using the ATSDR study’s identification of potential toxicity for the EGEs as support, DiScenza and Levine chose to include ethylene glycol in their research to develop sensitive and selective methods for detecting aliphatic alcohols [67]. ATSDR’s results helped the researchers prioritize ethylene glycol as one of the few analytes included in their efforts to develop high-throughput detection systems for aliphatic alcohols. That essentially made it easier to select chemicals by outlining possible health effects/metabolic pathways based on QSAR analyses. This work has further been used as evidence of the reliability of in silico methods [68,69], making researchers more likely to trust and use QSAR and other in silico methods. The decision tree developed and used in the EGE QSAR study to assign confidence in a prediction was also used by ATSDR researchers the following year. ATSDR researchers used it to investigate the joint toxicity of alkoxyethanols. QSAR modeling was used to identify chemicals that may interact with alkoxyethanols and alter their toxicity.

ATSDR has applied QSAR modeling techniques to address several areas of public health toxicology including exposure duration extrapolation as well as predicting the relative toxicity of breakdown products of military chemical agents. Such work has increased awareness by involving and encouraging collaborative efforts in the field of predictive toxicology. ATSDR researchers also used QSAR modeling to supplement their work on defining UFs for duration extrapolation in multiple chemical groups [70]. Researchers were able to use QSAR modeling to justify the exclusion of chemicals that were outliers as they disproportionately increased or decreased the average across-duration ratio for the chemical groups. QSAR modeling was thus able to yield UFs that were more specific than the traditional method of applying default UFs irrespective of the chemical in question. QSAR models also greatly assisted ATSDR researchers in the assessment of the toxicity of the breakdown products of sulfur mustard [71]. Using four different QSAR models, the researchers predicted median lethal dose (LD50) values for sulfur mustard and its potential breakdown products. LD50 is the dose at which 50% of the test population dies. ATSDR found that the breakdown products were potentially less toxic than the parent compound. This study exemplified the potential of QSAR modeling to assist with health guidance regarding chemical incidents, encouraging its widespread use to address various issues. QSAR modeling has thus allowed for notable toxicological discoveries and accomplishments at ATSDR and continues to progress, given our agency’s efforts to develop, evaluate, apply, and refine QSAR models and methods.

### 2.3. Computational Systems Biology

While research has established strong associations between exposures to certain chemicals and specific adverse outcomes, scientists have often been unable to designate causality due to a lack of mechanistic understanding. Computational systems biology integrates bioinformatics modeling, mathematics, high-throughput data, gene networks, protein interactions, and cellular processes to characterize potential linkages between environmental toxicants, biological pathways, and health outcomes [72,73,74,75]. This approach thus allows for substantiation of mechanistic hypotheses that can then be explored via experimentation.

As a part of a project employing computational systems biology modeling, ATSDR scientists investigated associations between three lipophilic persistent organic pollutants (TCDD, PCB 153, and *p*,*p*′-DDE) and metabolic diseases. They identified gene network pathways wherein the chemicals interacted to activate common downstream targets that may contribute to developing type 2 diabetes [76]. These findings were used by researchers exploring diabetes and diabetes-adjacent morbidities in an epidemiological study based in Spain that linked long-term exposure to persistent organic pollutants and the risk of metabolic syndrome [77]. Our findings were also used to emphasize the validity of investigating environmental toxicants in metabolic diseases in studies that assessed risk factors associated with diabetes, heart attack, and stroke among the Inuit Nation in Canada [78,79]. Based on our findings, Schulz and Sargis included TCDD in their review of evidence linking environmental endocrine-disrupting chemicals to the diabetes pandemic [80]. More recently, scientists at ATSDR led efforts to combine PBPK modeling, computational systems biology, and gene expression data to (1) discern the potential interaction mechanisms of toluene, ethylbenzene, and xylene (TEX) mixtures and (2) identify the health effects of long- and short-term exposure to the mixture [81].

### 2.4. Benchmark Dose (BMD) Modeling

Chemical risk assessment evaluates chemical toxicity and supports chemical registration, safety evaluation, and exposure limitation development. As a part of the risk assessment, a dose–response analysis determines the POD. The POD is the dose necessary to produce a particular effect of interest. Typically, researchers would identify the lowest observed adverse effect level (LOAEL) and corresponding no observed adverse effect level (NOAEL) to derive the POD. This process requires hypothesis testing to compare differences in response. However, the NOAEL/LOAEL approach does not consider the shape of the dose–response curve and is limited to experimental doses, dose spacing, and sample size of the study [82]. In contrast, the benchmark dose (BMD) methodology proposed by Crump [83] fits a mathematical model to all the dose–response data for the study’s endpoint. The BMD also incorporates experimental uncertainty in responses and allows users to assign a benchmark response appropriate for the assessment [84,85,86,87,88]. For these reasons, the US EPA [82] and EFSA [89] have advocated for BMD use for years.

Three software packages have been developed to promote the BMD modeling approach using continuous and dichotomous data from animal-based toxicology experiments: the US EPA’s BMDS 3.3.2 [90], the Netherlands National Institute for Public Health and the Environment’s PROAST software 71.1 [89], and Dream Tech’s Bayesian BMD 2.1.1 (BBMD) [91]. The major difference between these software packages is the algorithm. BMDS and PROAST have historically used maximum likelihood estimations of frequentist models, while BBMD uses the Bayesian statistical approach for parameter estimations of Bayesian models. Typically, BMDS chooses the ‘best’ model for BMD estimates based on the Akaike Information Criterion (AIC) value. However, recent BMDS versions now incorporate an alternative Bayesian model averaging approach for dichotomous data. This may be due to EFSA’s new guidance, which recommended changing from the frequentist to the Bayesian paradigm [92]. PROAST implements frequentist model averaging using the AIC as the basis for the weighting scheme. BBMD calculates the posterior BMD using the Bayesian model average (BMA) method.

In the past decade, BMD has moved from an emerging technology to an established technology at ATSDR. The software packages are more accessible, are available in various environments (websites, excel macros), and have more developed modeling capabilities. These advances in the understanding and accessibility of BMD modeling have greatly facilitated deriving MRLs. Over the past 15 years, ATSDR has revised older MRLs as additional studies have been performed and risk assessment approaches have been modified. In these revisions, BMD lower confidence limits, also known as BMDLs, were used if more appropriate. ATSDR analyzed the fold changes from the original MRLs to the updated MRLs. These findings suggest that using BMDLs as PODs does not cause a clear bias toward decreasing or increasing MRL values. Additionally, BMDL analysis does provide a more quantitative approach to analyzing dose–response data and incorporating experimental uncertainty.

### 2.5. Fate and Transport Modeling

It is often difficult for public health professionals to access direct measures of exposure and dose, especially for historical exposures. ATSDR introduced the Exposure-Dose Reconstruction Program (EDRP) to address this issue. EDRP was an interdisciplinary team. Fate and transport models are important tools for predicting contaminant exposures [93,94,95,96,97,98,99,100,101,102,103]. Exposure-dose reconstruction involves the use of computational models and tools to provide estimates of the concentration of toxic substances in people who may be at risk of exposure to chemicals from hazardous waste sites. Environmental fate and transport modeling and water-distribution system modeling were introduced as a part of EDRP in 1993 and are now a part of OIA/SSS. They greatly augment the agency’s capacity to evaluate exposures and doses, improving the subsequent health assessments and studies.

#### Water Modeling: Reconstruction of Historical Drinking Water Contamination

Between 1950 and 1985, the drinking water at U.S. Marine Corps Base Camp Lejeune was contaminated with VOCs. In recent years, ATSDR scientists sought to evaluate the association between exposure to the contaminated water and various birth outcomes, and they have published multiple papers describing their reconstruction of the historical contamination. One case–control study focused on birth defects and childhood cancers. Using groundwater fate and transport and water-distribution system models to assess historical exposures, the researchers found certain water contaminants were associated with neural tube defects [104]. In another study, the researchers found that these contaminants were associated with adverse effects such as preterm birth and fetal growth retardation [105].

Exposure-dose modeling was also used to evaluate adult mortality and cancer outcomes. Studies found elevated hazard ratios at Camp Lejeune for death from several causes, including cancers and neurodegenerative diseases in marine and naval employees [106] and civilian employees [107]. The confidence intervals were wide for most hazard ratios as only 14% of the cohort had died by the end of follow-up, resulting in small numbers of cause-specific deaths. Furthermore, another study suggested possible associations between male breast cancer and being stationed at Camp Lejeune and cumulative exposure to several VOCs [108]. However, the confidence intervals for the hazard ratios were wide.

Ongoing applications of ATSDR’s exposure-dose reconstruction modeling focused on per- and polyfluoroalkyl substances (PFASs) in the following projects:

- Pease International Tradeport of Portsmouth, New Hampshire
  The historical reconstruction used a materials mass balance model to compute flow-weighted average concentrations of PFASs in public drinking water.
- PFAS Exposure Assessments
  Evaluated 50+ PFAS sites to determine the nationwide representation of concentrations of PFAS in drinking water.
- PFAS Multi-Site Study
  Historical Reconstruction Workgroup oversees fate and transport analyses and water-distribution system analyses to estimate concentrations of PFAS in drinking water. Estimates will be used as input for PBPK modeling.
- Saint-Gobain (Merrimack, NH)
  Historical reconstruction of concentrations of PFASs in the public drinking water.
- NASA Wallops Flight Facility (Town of Chincoteague, VA)
  Historical reconstruction of concentrations of PFASs in the public drinking water.
- Warminster and Willow Grove, Pennsylvania
  Historical reconstruction of concentrations of PFASs in the public drinking water.

## 3. Collaborations

ATSDR’s progress in simulation sciences has been extensive in the last decade. However, these modeling efforts would not have been as successful without support and collaboration from other CDC/ATSDR teams and the projects ATSDR works on. The projects involve inhalation toxicity modeling, surveillance, biomonitoring reports, and literature reviews. The technical support and expertise provided by these scientific researchers are crucial in driving the field of simulation science forward. The research integrates epidemiological and toxicological approaches, and the collaboration demonstrates how multidisciplinary teams can use their combined skills to achieve a common goal.

### 3.1. Internal Collaborations

#### 3.1.1. Inhalation Toxicity and Emergency Response

Acute, short-term exposures to airborne chemicals are an ongoing threat to public health, especially in emergency response situations. However, there are challenges to determining HGVs for inhalation toxicity. One of the main issues is that certain durations of exposure may not have experimental data. Subsequently, extrapolation modeling is crucial for determining health guidance. Modeling inhalation toxicity is challenging because the adversity of health effects is a two-dimensional problem: it depends on the inhalant concentration and the duration of exposure. The simplest modeling assumption would be that the toxic effect is proportional to the product of inhalant concentration and duration of exposure. However, this equation does not hold for many volatile compounds. Thus, a more complex concept of toxic load was introduced: the toxic load equation (TLE), which is chemical-specific [109].

While short-term exposure recommendations may exist for certain chemicals of interest, these recommendations are sometimes limited to specific exposure periods. This provides inadequate guidance for exposure that may last longer or shorter periods. Demchuk et al. used QSAR/TLE modeling to derive HGVs for dimethyl sulfide using data reviewed by the American Industrial Hygiene Association [110]. The HGVs were in good agreement with the American Industrial Hygiene Association’s 1 h health guidance and were able to extend the guidance to durations ranging from 10 min to 8 h.

Another issue is that inhalation experiments require large numbers of animals to model the concentration and duration confidently. This requires a method that combines experimental data from multiple studies. Prussia et al. achieved this by conducting a meta-analysis wherein 28 volatile chemicals were investigated [111]. Data evaluation rules were derived from the meta-analysis and structured into a decision tree. The tree provides an unambiguous framework for TLE derivation from probit data of any kind and greatly facilitates inhalation risk assessment. The tree uses all of the appropriate experimental studies to arrive at an exposure threshold for the chemical, increasing the precision and scientific rigor of its public health assessment.

#### 3.1.2. Biomonitoring, Surveillance, and Literature Reviews

Biomonitoring data provide invaluable information for identifying trends and conducting further research and analysis on the target population. Simulation science researchers provide technical support to other projects by agency researchers. ATSDR researchers analyzed NHANES data from 2007 to 2012 to identify and characterize prevalent cadmium, mercury, lead, and arsenic combinations in the general U.S. population [112]. The study was intended to provide a solid basis for future work on the four toxic metals and their combinations. Similarly, simulation science researchers provided technical consultation to epidemiologists at ATSDR who used biomonitoring data for exposed communities. Such work, in turn, supports ongoing attempts to develop simulation science models for chemical mixtures and to better understand potential biological pathways.

While collaborations are necessary to amalgamate the expertise needed to develop and improve simulation science approaches, assessing the existing knowledge base and identifying relevant gaps are equally important in developing and enhancing simulation science methods. Literature reviews, although laborious, provide an abundance of information that is distilled to the most relevant components. For example, an expert review by ATSDR researchers on PCB exposures and health effects [113] has been instrumental in supporting research to understand the toxic potential of PCBs. That review provided substantial detail on different health effects, including nephrotoxicity, developmental effects, and cancer. More importantly, the paper discussed both human and animal studies, which enabled a better understanding of the relationship between the exposure and toxicity of PCBs.

Another important method for identifying patterns or issues to address, and one of CDC’s principal functions, is public health surveillance. The National Toxic Substance Incidents Program (NTSIP) is a surveillance initiative created by the ATSDR. NTSIP collects data on acute toxic substance incidents and their association with mortality and morbidity in terms of public health. The program collected data in nine states (California, Louisiana, North Carolina, New York, Missouri, Oregon, Tennessee, Utah, and Wisconsin). The large set of data helps identify patterns and causes of incidents. Furthermore, NTSIP can provide valuable information to those working to prevent or respond to similar incidents. In addition to supporting public health professionals’ characterized occurrences of acute toxic substance releases, one publication was used to highlight the danger of such chemical incidents. Hu et al. aimed to analyze the pollution inequality of toxic chemical releases across regions and income groups in the U.S. [114].

ATSDR’s great strides in simulation sciences have not been achieved in a vacuum. The concurrent research and projects within and beyond the CDC/ATSDR continue to fuel and be fueled by the progress of simulation sciences in public health.

### 3.2. External Collaborations

Scientists from the Simulation Science Section at ATSDR have been involved in efforts to further simulation science approaches in various industries. Efforts have been made to meet the needs of stakeholders such as other federal agencies, academia, and the private sector.

#### 3.2.1. Interagency Collaborations

Several recent interagency efforts promoted the use of simulation science methods, such as PBPK and QSAR modeling, and were led by the Interagency Coordinating Committee on the Validation of Alternative Methods (ICCVAM) [115]. The group’s main aim is to reduce the use of lab animals during toxicity testing of chemicals while optimizing the use of scientific methods and knowledge. One of the premier ways of minimizing the use of test animals is through validating and promoting alternative methods, such as computational toxicology methods, that move research from observational to predictive science. ICCVAM’s Strategic Roadmap [116] highlights how workgroups comprising national and international participating agencies could encourage confidence in using and developing new methods. ATSDR is a part of several ICCVAM workgroups, including the In Vitro to In Vivo Extrapolation workgroup (IVIVE WG), the Read-Across workgroup (RAWG), the Acute Toxicity workgroup (ATWG), and the Verification workgroup (VW). These interagency collaborations support a more transparent understanding of the progress and future work potential in the field of simulation sciences. The agency representatives can convey the needs and problems faced by their organizations precisely and unambiguously.

ATSDR was also a part of the Collaborative Modeling Project for Androgen Receptor Activity (CoMPARA). In the collaborative, scientists from 25 international research groups generated QSAR models for androgen receptor activity and screened many chemicals. The project produced consensus model predictions [117]. This project aimed to combine the strengths of multiple models to generate consensus predictions about the toxicology of large numbers of chemicals. The researchers hoped the predictions would further the quest to optimize high-throughput virtual screening methods. Ultimately, the project aided the creation of open-source tools that the scientific community can apply for various purposes.

ATSDR initiated a series of Interagency Computational Toxicology Colloquia (ICTC), which included participants from ATSDR, FDA, EPA, the National Institutes of Health (NIH), Chemical Genomics Center (NCGC), National Institute of Environmental Health Sciences (NIEHS), and other interested agencies. Following the ICTCs, ATSDR and the National Center for Toxicological Research (FDA-NCTR) developed a joint funding initiative in hepatotoxicity. Conventionally, hepatotoxicity has been investigated based on extensive traditional toxicology testing methods. Traditional methods often involve a detailed, comprehensive study under various doses. These studies are costly, time-consuming, and ineffective for extensive chemical screening and prioritization. Therefore, considerable effort has been directed toward alternative methods, including high-throughput molecular and cell-based assays and computational modeling techniques. As a part of these endeavors, FDA-NTCR and ATSDR developed highly efficient QSAR and spectral data–activity relationship models to identify chemical hazards to the human liver [118]. The models provide additional novel in silico tools for identifying hepatotoxicity more effectively.

Furthermore, ATSDR contributed to the efforts of an external workgroup funded by the Health & Environmental Sciences Institute (HESI) that produced a PBPK model reporting template [119]. Tan et al. designed the template to facilitate the review process when submitting PBPK models to regulatory agencies. In addition to improving the chances of the model’s acceptance for regulatory purposes, the template can educate others on best practices for planning to use and communicating about a new model.

#### 3.2.2. Academic Collaborations

Scientists at ATSDR also work with university researchers to facilitate research goals using QSAR modeling. A 2019 study by Uwimana et al. sought to identify the isoforms of an enzyme (cytochrome P450) that metabolized a group of polychlorinated biphenyls (PCBs) to their hydroxylated forms [120]. ATSDR used in silico methods, specifically ADMET Predictor and MetaDrug, to predict the isoforms that could metabolize the PCBs to OH-PCBs. This process allowed the researchers to perform in vitro experiments using a narrowed set of enzymes, significantly reducing the time and cost required to perform the experiments and helping focus the scope of the work.

Similarly, ATSDR has been involved in a series of studies investigating the metabolites of certain individual PCBs by combining in silico and in vitro methods to optimize the research process. In 2020, Zhang et al. studied the metabolomics of PCB 11 (3,3′-dichlorobiphenyl) using ADMET Predictor and MetaDrug to predict PCB 11’s metabolites. The predictions were aligned to a significant extent with what was previously found in rat studies. The team predicted 4 of the 30 metabolites detected in the in vitro portion of the study [121]. Subsequent studies on the P450-mediated biotransformation of lower chlorinated PCBs such as PCB 3 (4-chlorobiphenyl) and PCB 2 enhanced the preliminary research process by employing ADMET Predictor and MetaDrug.

Another noteworthy collaboration was between researchers across the U.S. (ATSDR), Australia, Thailand, and Japan. The collaboration resulted in a study on Thai subjects that investigated and established a link between cadmium toxicity markers and clinical kidney function measures [122]. Satarug and colleagues were able to reassert a previously dismissed role of cadmium exposure in kidney toxicity: they discovered an association between cadmium and chronic kidney disease (CKD). This work was a part of an undertaking to model cadmium toxicity by comparing low- and high-exposure populations [24].

In silico predictive methods can transform scientific research by identifying a short list of variables or candidates for further inquiry. Yet, such simulation science methods do not have widespread use, making collaborative work crucial in catalyzing change. Scientists trained in silico methods and tools can help educate colleagues who were trained in different fields and who are interested in simulation science.

#### 3.2.3. Private Collaborations

A lack of standardized guidance regarding the use and interpretation of in silico methods is one of the major reasons they are not widely used and heavily relied upon. While several agencies and organizations have developed their own sets of protocols, there remains a need for a universal set of guidance or standardized protocols that scientists across different fields can rely upon. Working towards this goal, ATSDR has been a part of an international consortium that brings together representatives from various fields to outline a framework for developing in silico toxicology protocols for major endpoints of interest [123]. While the initial publication by Myatt et al. laid out the schematics for developing in silico toxicology protocols (QSAR approaches), the specific endpoints were, and continue to be, addressed in publications through the work of focused subgroups formed by the consortium participants.

The inter-industry nature of the collaboration is essential for developing universally accepted standardized protocols. Representatives from various fields can convey their needs and requirements and appreciate the expertise contributed by the other members. The amalgamation of different perspectives warrants that, once the in silico protocols have been established, users will not have reservations about the applicability of those guidelines to their work. To advance this overarching goal, ATSDR has collaborated with various stakeholders in the pharmaceutical industry to delineate the current status and future needs for using in silico methods to predict and assess heart, kidney, and lung toxicities [124]. The publication they produced provides a foundation for developing in silico protocols for organ toxicities by outlining the current methods and the associated issues and data gaps. Crofton and colleagues also published a paper following a similar framework on using in silico methods in neurotoxicity hazard assessment [125]. The publication emphasizes the utility of combining computational and experimental methods and provides a draft assessment framework for a standardized in silico toxicology protocol for neurotoxicity. As proposed by in silico methods, the likelihood of off-target effects could be better evaluated by improved high-throughput screening methods. These methods would improve both awareness and guidance with regard to new and old pharmaceutics. ATSDR is actively providing expert support and guidance via collaborations with several companies in the private sector to bring these efforts to fruition.

## 4. Challenges and Future Directions

Computational approaches have revolutionized the field of toxicology and risk assessment, offering several advantages. First, they enable rapid and cost-effective screening of chemicals for potential toxicity, which reduces the need for time-consuming and expensive animal testing. Second, computational models can predict toxic effects at various levels of biological organization. This can provide valuable insights into mechanisms of toxicity and aids in risk assessment. Additionally, these approaches offer a systematic way to integrate diverse data sources, including chemical structures, molecular properties, ADME, and biological pathways, to identify new hazard endpoints [81].

**Figure 2:**
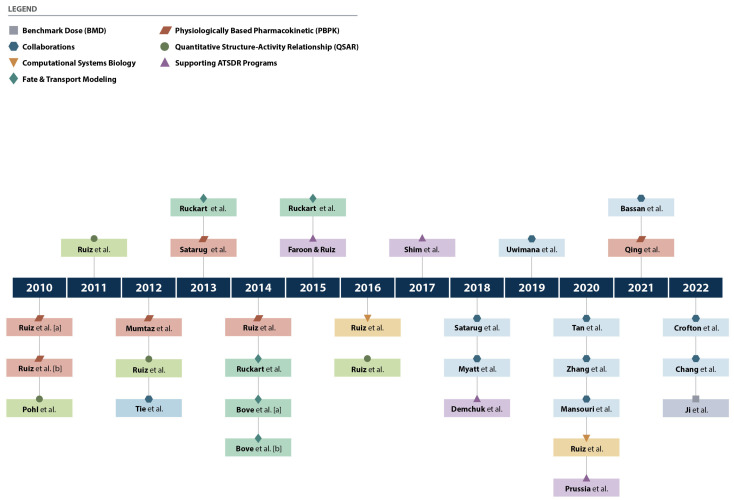
Timeline of publications [19,20,24,25,26,32,40,66,70,71,76,81,104,105,106,107,108,110,111,112,113,117,118,119,120,121,122,123,124,125,126,127].

Multiple manuscripts have been published describing ATSDR’s work, research interests, and collaborations in simulation science over the past decade. Figure 2 shows the details of the work published by ATSDR scientists and the application of computational tools and links them with published citations. It shows the consistent application of PBPK modeling, QSAR modeling, computational systems biology, benchmark dose modeling, and fate and transport modeling during the past decade at ATSDR. PBPK and QSAR modeling has been the most published of these computational modeling approaches, while benchmark dose modeling has been the least published by ATSDR scientists yet is regularly used to support internal ATSDR risk assessments. With the advance of time, ATSDR publications have been recognized, and ATSDR collaboration in several efforts has increased during the past five years.

**Figure 3:**
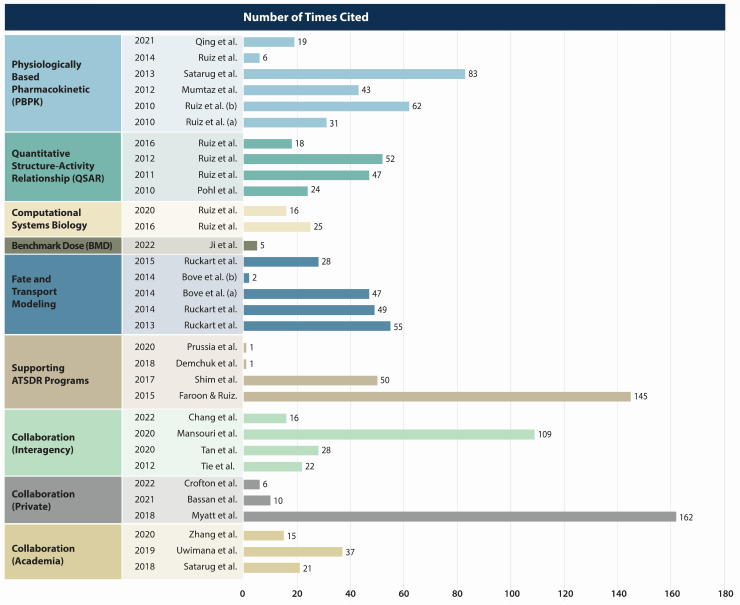
Publication impact: number of citations [19,20,24,25,26,32,40,66,70,71,76,81,104,105,106,107,108,110,111,112,113,117,118,119,120,121,122,123,124,125,126,127].

Figure 3 clearly illustrates the impact of ATSDR’s publications, as measured by the number of references cited in the Google Scholar databases as of July 2023. Google Scholar is a freely accessible product of Google; it collects citation and reference information using web crawlers that roam through websites containing scholarly information. These data show the relevance and influence of ATSDR’s work in computational modeling. There were nine publications on PBPK modeling, ten on QSAR modeling, three on computational systems biology, one on BMD modeling, five on fate and transport modeling, six on supporting ATSDR programs, and fifteen in collaborations (seven interagency collaborations, three private collaborations, and five collaborations in academia). The PBPK publications appear to have had the most impact on computational modeling development and applications, followed by QSAR modeling and fate and transport modeling [119,123].

The most cited publication was the in silico modeling, with 199 citations (Figure 3). ATSDR’s participation in consortia and collaboration with national and international working groups had a significant impact. The most cited publications are related to model development, and model application of computational modeling based on PBPK and QSAR modeling were the next most cited. The significance of integrating multiple computational toxicology modeling approaches and PBPK modeling, computational systems biology, and fate and transport modeling that interpreted biomonitoring and epidemiological data are also well represented, as seen in the citations. The details of support lent to other ATSDR programs by simulation science publications are also described in this figure.

The future of computational modeling at ATSDR holds significant promise for advancing public health and human risk assessment. One key area of focus is the integration of emerging technologies, such as artificial intelligence (AI) and machine learning, to enhance the predictive capabilities of PBPK models [128,129,130,131,132,133,134,135,136]. In the future of simulation science, AI could address public health concerns by supporting spatial modeling, risk and disease prediction, and public health surveillance [137]. However, since the application of AI is a currently evolving topic, future implementation at ATSDR will need to consider development of infrastructure, training people to improve technical understanding of applications and pitfalls, addressing the need for more data, and dealing with ethical and privacy issues. This strategic direction aligns with the organization’s commitment to harnessing advanced algorithms and vast amounts of data to provide more accurate predictions on environmental chemical distribution, metabolism, and elimination in diverse populations.

Additionally, there is a growing emphasis on incorporating genetic variability and probabilistic risk assessment into PBPK models to better understand individual susceptibility to chemical toxicity or adverse health effects, which will address the issues of sensitive populations and environmental justice. These advancements in PBPK modeling can potentially revolutionize public health interventions by enabling personalized risk assessment approaches and improving risk assessment strategies for environmental chemicals.

QSAR modeling has significantly advanced in predicting the potential toxicological effects of chemicals on human health. However, several limitations and challenges hinder its application in public health and human risk assessment. A major limitation is the lack of comprehensive databases containing high-quality toxicity data. This limits the accuracy and reliability of QSAR models. Additionally, QSAR models often do not account for complex biological processes and interactions within the human body, resulting in uncertainties when extrapolating results to real-life scenarios. Moreover, there is a need for standardized protocols and guidelines to ensure consistency and reproducibility across different QSAR studies. Addressing these limitations and challenges will be crucial for improving the effectiveness and reliability of the use of QSAR models in public health and human risk assessment. With the increasing need for accurate risk assessment in public health, there has been a continuous effort to enhance the capabilities of QSAR models. Recent advances have focused on improving these models’ accuracy, reliability, and applicability. One significant advancement is the integration of big data analytics and machine learning algorithms into QSAR modeling. This allows researchers to analyze vast amounts of chemical data and make more precise predictions of toxicity and exposure levels.

Another computational approach, benchmark dose modeling, plays a critical role in evaluating the potential toxicity of chemicals and assessing human health risks associated with chemical exposure. Advances in digital imaging, cell painting, and histopathology data are now being explored for integration in dose–response analyses; however, it is important to address potential risks when using extrapolated BMDL values outside of measured exposure ranges [138,139,140]. BMD results should be retained for further consideration when (1) the difference between the estimated BMD and its BMDL is not greater than 20 times and (2) the estimated BMDL value is not less than 10 times the lowest non-zero dose level [141]. Benchmark dose modeling can produce unrealistic BMD and BMDL values for certain dose–response curves, particularly when the data provide little useful information about the dose–response relationship [90]. The ideal solution is to collect additional data in the dose range that the current studies have missed. If this is not possible and modeling will not yield useful results, the NOAEL value is used as the POD, although the data gaps and inherent limitations of that approach should be acknowledged [90]. Traditional animal-based toxicology experiments are labor-intensive and time-consuming and lack explanations for mechanisms. Chemical risk assessment is transitioning from an in vivo observational to in vitro functional science through the use of omics technologies, named toxicogenomics, for HTS [126,142,143]. The evolving landscape of HTS technologies and New Approach Methodologies (NAMs) is driving the transition of the Tox21 initiative towards more resource-efficient methods. With the U.S. EPA formerly set to eliminate all testing in mammals by 2035 and the European Union banning animal testing on cosmetic ingredients and finished products since 2013, the importance of benchmark dose modeling and simulation science in these areas is amplified [144,145,146]. As we work toward ATSDR’s mission, the future of the simulation science team in benchmark dose modeling will be centered around leveraging the advancements in HTS and NAMs to enhance the accuracy, efficiency, and cost-effectiveness of chemical toxicity evaluations.

Integrating PBPK/IVIVE models with benchmark dose modeling for in vitro data is where simulation science can significantly contribute. These integrated models can bridge the gap between in vitro and in vivo responses, provide valuable insights into chemical exposures, and facilitate the refinement of risk assessments [147,148,149,150,151,152,153]. Moreover, applying the benchmark modeling approach to epidemiological data and its potential use in interdisciplinary fields, such as interindividual variability assessment, offers promising avenues for future research. The Simulation Science Section can collaborate with epidemiologists and other experts to explore and refine these methodologies, contributing to a more comprehensive understanding of human health risks.

Recent research in computational systems biology has provided an innovative in silico strategy to integrate computational systems biology, AI, and machine learning models that may help to understand and propose mechanisms of action and adverse outcome pathways, offering new insights in the interpretation of the interaction between chemical exposure, genes, and diseases [154,155,156,157].

Environmental fate and transport modeling and water modeling play an integral role in estimating the cumulative exposure of hazardous substances internalized by persons expected to have come into contact with substances associated with hazardous waste sites [95,96,101,104]. ATSDR estimates past exposures and uses population information and biological sampling to determine current exposure levels. For example, modeled drinking water concentration outputs that estimate past and current human exposure can be integrated with PBPK models and used as inputs to help better determine human health risks.

ATSDR is often involved in chemical emergencies and assessments of potential toxic effects. Thus, building capacity in inhalation toxicity modeling and hazard forecasting is an important aspect of the agency’s mission. However, achieving these goals is challenging. Firstly, experimental data for TLE development are only available for a handful of volatile hazardous chemicals. Therefore, the number of quality HGVs is limited. Research is necessary to fill these data gaps, perhaps by leveraging other computational methods such as in silico and pharmacokinetic modeling.

Industrial processes continually evolve, causing changes in the spectrum of chemical pollution. According to the EPA Chemical Data Reporting (CDR) Program, the use of chemicals has increased almost linearly in recent decades. There are approximately 300 new chemicals added annually, reaching 8649 chemicals in 2020 [158]. Therefore, more chemicals may deserve risk assessment reviews and chemical-specific HGVs. For example, there are more than 20 isocyanate compounds on the 2020 CDR list, and many of these compounds have not undergone comprehensive inhalation exposure risk assessments, and none have HGVs developed. Thus, vast unexplored areas in inhalation toxicity modeling deserve public health attention and further development.

Collaboration and data sharing are crucial to advance simulation science research at ATSDR. By fostering collaboration among scientists, industry professionals, and regulatory agencies, ATSDR aims to leverage collective expertise and resources to improve the assessment of chemical hazards. Through collaborative efforts, researchers can share computational models, tools, and databases that aid in predicting toxicity [117,127]. This will enable a more comprehensive evaluation of potential health risks associated with various chemicals. By pooling resources, diverse datasets can be integrated into computational models, enhancing their accuracy and applicability. Moreover, data-sharing initiatives ensure transparency, acceptance, and reproducibility of methods in simulation science research. Risk assessors and scientists can access shared datasets to validate findings or build upon the existing knowledge. This facilitates the development of standardized approaches that promote consistency across studies [119,123].

## 5. Conclusions

The Agency for Toxic Substances and Disease Registry uses computational modeling to assist state and local health departments, federal agencies, academia, and public health professionals. Specifically, ATSDR employs computational tools to support the investigation and understanding of health effects from exposure to environmental chemicals. Simulation science continues to evolve. Researchers are identifying new data sources and applying cutting-edge modeling approaches to understand and interpret human chemical exposure.

ATSDR’s simulation science methods and tools provide the information needed for the following tasks:
  - Interpreting toxicological data for site- and chemical-specific health consultations and exposure assessments;
  - Interpreting emergency response activities;
  - Interpreting applied toxicology research;
  - Developing toxicological profiles;
  - Assessing and filling chemical-specific data needs.

As research and technology advance rapidly, the promise of reducing the adverse effects from exposure to chemicals on human health will become more attainable. This will, in turn, expand the potential applications of simulation science approaches to benefit the environment and human health. We intend to advance the growth of simulation science approaches to support environmental and public health protection by promoting the standardization of these methods as well as encouraging continued multidisciplinary collaborative work in the field.

## Data Availability

Data are contained within the article.
